# Inhibition of mitoNEET attenuates LPS-induced inflammation and oxidative stress

**DOI:** 10.1038/s41419-022-04586-2

**Published:** 2022-02-08

**Authors:** Seunghee Lee, Byeong Geun Seok, Seon-Jin Lee, Su Wol Chung

**Affiliations:** 1 School of Biological Sciences, College of Natural Sciences, University of Ulsan, 93 Daehak-ro, Nam-gu, Ulsan, 44610 South Korea; 2 Genome Structure Research Center, Korea Research Institute of Bioscience and Biotechnology, Yuseong-gu, Daejeon, 305–806 South Korea

**Keywords:** Mechanisms of disease, Stress signalling, Sepsis, Bacterial infection, Target identification

## Abstract

MitoNEET (mitochondrial protein containing Asn–Glu–Glu–Thr (NEET) sequence) is a 2Fe–2S cluster-containing integral membrane protein that resides in the mitochondrial outer membrane and participates in a redox-sensitive signaling and Fe–S cluster transfer. Thus, mitoNEET is a key regulator of mitochondrial oxidative capacity and iron homeostasis. Moreover, mitochondrial dysfunction and oxidative stress play critical roles in inflammatory diseases such as sepsis. Increased iron levels mediated by mitochondrial dysfunction lead to oxidative damage and generation of reactive oxygen species (ROS). Increasing evidence suggests that targeting mitoNEET to reverse mitochondrial dysfunction deserves further investigation. However, the role of mitoNEET in inflammatory diseases is unknown. Here, we investigated the mechanism of action and function of mitoNEET during lipopolysaccharide (LPS)-induced inflammatory responses in vitro and in vivo. Levels of mitoNEET protein increased during microbial or LPS-induced sepsis. Pharmacological inhibition of mitoNEET using mitoNEET ligand-1 (NL-1) decreased the levels of pro-inflammatory cytokines such as IL-1β, IL-6, and TNF-α in animal models of sepsis, as well as LPS-induced inflammatory responses by macrophages in vitro. Inhibition of mitoNEET using NL-1 or mitoNEET shRNA abrogated LPS-induced ROS formation and mitochondrial dysfunction. Furthermore, mitochondrial iron accumulation led to generation of LPS-induced ROS, a process blocked by NL-1 or shRNA. Taken together, these data suggest that mitoNEET could be a key therapeutic molecule that targets mitochondrial dysfunction during inflammatory diseases and sepsis.

## Introduction

Inflammation is critical for healing, but uncontrolled and dysregulated inflammation can increase the risk of developing various diseases [1]. Sepsis, caused mainly by bacterial infection, is a highly inflammatory disorder that, in severe cases, can cause organ dysfunction and death [2]. The link between sepsis-associated organ failure and mitochondrial dysfunction is increasing interest to researchers [3]. Sepsis-induced mitochondrial dysfunction mediates hyperinflammation through cellular metabolic disorders, insufficient energy production, and oxidative stress; as such, it plays a key role in the development of sepsis-related multiorgan failure [4–6]. Mitochondria, dynamic organelles that serve as the power house of the cell, are a major source of reactive oxygen species (ROS); they are also the site where iron is transformed into its bioactive form [7–10]. Increased mitochondrial iron accumulation due to pro-inflammatory signaling promotes oxidative damage by catalyzing generation of ROS and causing mitochondrial dysfunction [9, 11]. These processes develop into a vicious inflammatory cycle [12]. Therefore, the mitochondrial iron level must be strictly regulated to avoid iron-mediated damage and maintain mitochondrial function. Several studies demonstrate that targeting mitochondrial iron accumulation using iron chelators has the potential to improve the prognosis of sepsis [8, 13, 14].

The mitochondrial protein mitoNEET containing Asn–Glu–Glu–Thr (NEET) sequence (also referred to as CDGSH (C–X–C–X2–(S/T)–X3–P–X–C–D–G–(S/A/T)–H) iron sulfur domain 1 (CISD1)) is a 2Fe–2S cluster-containing, redox-sensitive protein that resides on the outer mitochondrial membrane; as such, it is a powerful regulator of mitochondrial iron content [15–17]. Only when the mitoNEET [2Fe–2S] clusters are oxidized do they transfer [2Fe–2S] clusters to apo-proteins and electrons from FMNH2 (reduced 1,5-dihydro form of flavin mononucleotide) to oxygen or ubiquinone in mitochondria [18–20]. Therefore, mitoNEET exerts marked effects on cellular and systemic metabolic homeostasis by acting as a powerful regulator of mitochondrial iron content. Early studies showed that mitoNEET plays a key role in regulating cellular energy use, lipid metabolism, and cancer cell proliferation and tumor formation [21–23]. Recent studies on the effects of redox regulation by mitoNEET demonstrate that mice overexpressing mitoNEET exhibit reduced ROS generation by mitochondria; however, oxidative phosphorylation and electron transport are significantly upregulated in the absence of mitoNEET [6, 24]. This is associated with generation of ROS by mitochondria, along with mitochondrial dysfunction [25]. Thus, mitoNEET is involved in a variety of human pathologies, including cystic fibrosis, diabetes, muscle atrophy, and neurodegeneration [25–27].

Initially, mitoNEET was identified as a mitochondrial target of thiazolidinediones such as pioglitazone and rosiglitazone, a peroxisome proliferator-activated receptor gamma (PPAR-γ) agonist, a class of medicines used to treat type-2 diabetes [28, 29]. Thiazolidinediones show antioxidative and anti-inflammatory activity in different disease models, including sepsis [30–32]. Overproduction of ROS during sepsis is thought to be a central part of the disease process [6, 33]. However, the role of mitoNEET in sepsis is unknown. Here, we have used a mitoNEET ligand (NL-1), modified TZD as a weaker affinity for PPARγ [15, 28, 34] and show that inhibiting expression or activity of mitoNEET reduces inflammation and oxidative stress during inflammatory responses and sepsis.

## Results

### Inflammatory stimuli induce expression of mitoNEET during sepsis

**Fig. 1:**
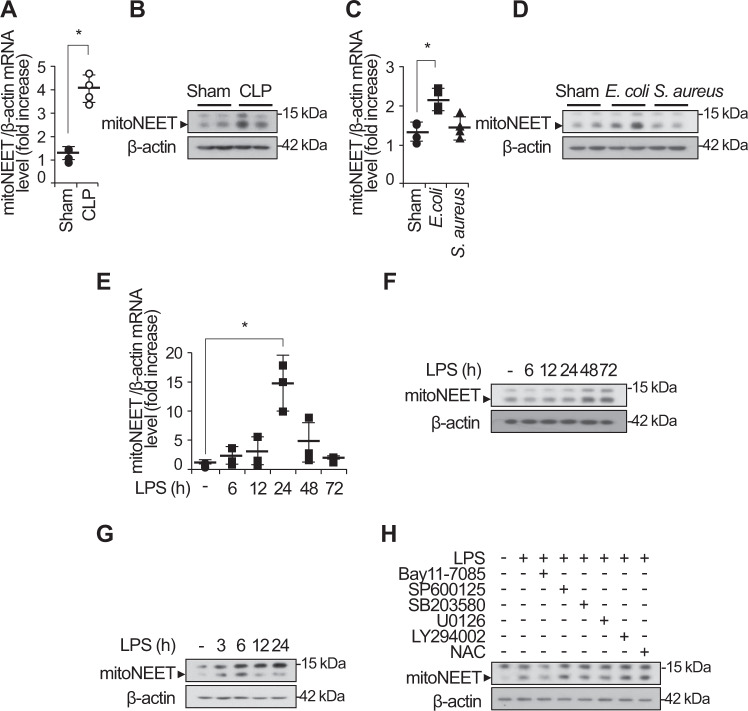
Expression of mitoNEET mRNA and protein increases during microbial sepsis. Total RNA and protein were extracted from the spleen 48 h after sham or CLP surgery, and fibrin clot-induced microbial sepsis was triggered by *E. coli* or *S. aureus* bacteria (1 × 10^8^ CFU). Expression of mitoNEET mRNA (**A, C**) and protein (**B, D**) levels was assessed by quantitative real-time RT-PCR or western blotting. *P < 0.05 for sham vs. CLP or fibrin clot-induced microbial sepsis. C57BL/6 mice were injected with LPS (20 mg/kg) or vehicle, and total RNA and protein were extracted from the spleen 6, 12, 24, 48, and 72 h after administration of vehicle or LPS (100 ng/mL). Total protein was extracted from BMDMs 3, 6, 12, and 24 h after administration of vehicle or LPS (100 ng/mL). Expression of mitoNEET mRNA (**E**) and protein (**F, G**) was assessed by quantitative real-time RT-PCR or western blotting. Total protein was extracted from BMDMs 6 h after administration of vehicle or LPS (100 ng/mL) plus a signaling inhibitor (5 µM BAY11-7082, 10 µM SP600125, 10 µM SB203580, 10 µM U0126, 10 µM LY2940002, or 20 mM NAC). Expression of mitoNEET protein was assessed by western blotting (**H**). All data are expressed as the mean ± SD from three independent experiments. *P < 0.05 for vehicle vs. LPS treatment.

Mitochondrial damage or dysfunction is the major cause of the multiple organ failure during sepsis [33]. Prior studies show that mitoNEET, an outer mitochondrial membrane protein, plays an important role in regulating mitochondrial function, especially oxidative capacity [25, 31, 35]. In this study, we hypothesized that mitoNEET plays a role in inflammation and oxidative stress during sepsis. To identify the role of mitoNEET during sepsis, we assayed expression of mitoNEET after induction of sepsis. Wild-type mice on a pure C57BL/6 genetic background were subjected to cecal ligation and puncture (CLP) to induce polymicrobial peritonitis, bacteremia, and sepsis. We then examined expression of mitoNEET mRNA and protein in the spleen 48 h later (Fig. 1A, B). Expression of mitoNEET mRNA and protein increased significantly (by 4.3-fold (Fig. 1A) and 4.1-fold (Fig. 1B), respectively) in mice with CLP-induced sepsis compared with sham mice (*n* = 4 per group). In addition, expression of mitoNEET mRNA and protein increased by 2.2-fold (Fig. 1C) and 3.4-fold (Fig. 1D), respectively, in mice with Escherichia coli (Gram-stain negative)-induced sepsis compared with sham mice (*n* = 4 per group). These data suggest that mitoNEET plays a critical role in sepsis caused by Gram-negative bacteria. To investigate the significance of mitoNEET induction by Gram-negative bacteria, we assessed its mRNA and protein levels in the spleen after induction of lipopolysaccharide (LPS)-mediated sepsis. LPS is a common pathogenic component of the outer membrane of Gram-negative bacteria. We harvested total RNA and protein from the spleen at 6, 12, 24, 48, and 72 h after LPS injection. We found that mitoNEET mRNA levels began to increase by 6 h after LPS injection, and marked induction of mitoNEET was evident at 24 h (compared with vehicle) (Fig. 1E); protein levels increased at 48 h (Fig. 1F). Furthermore, expression of mitoNEET protein in bone marrow-derived macrophages (BMDMs) increased in the presence of LPS compared with vehicle (Fig. 1G). To identify the signaling pathway involved in regulating mitoNEET expression in LPS-stimulated macrophages, we used specific inhibitors Bay 11-7085 (an NF-kB inhibitor), SP600125 (a JNK MAP kinase inhibitor), SB203580 (a p38 MAP kinase inhibitor), U0126 (a mitogen-activated protein kinase kinase 1/2 (MEK1/2) inhibitor), LY294002 (a PI3 kinase inhibitor), and NAC (N-acetyl-L-cysteine, cytosolic ROS scavenger, A7250). BMDMs were treated with these kinase inhibitors in the presence of LPS, and levels of mitoNEET protein were assessed 6 h later. Bay 11-7085 blocked LPS-induced mitoNEET expression (Fig. 1H); however, the other inhibitors had no effect. These data suggest that mitoNEET may have a critical role in inflammation during sepsis, and that the LPS-induced NF-kB signaling pathway is involved in induction of mitoNEET expression under inflammatory conditions.

### Inhibition of mitoNEET reduces inflammatory responses during LPS stimulation of macrophages

**Fig. 2:**
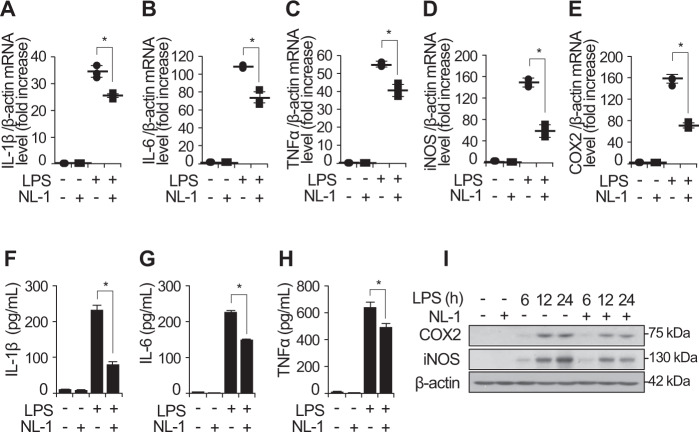
Expression of inflammatory mediators decreases in the presence of the mitoNEET inhibitor NL-1. Total RNA, cell supernatants, and total protein were harvested from RAW264.7 cells 12 h (for mRNA) and 24 h (for protein) after treatment with vehicle, NL-1 (20 µM), LPS (100 ng/mL), or LPS plus NL-1 (20 µM). Levels of mRNA encoding pro-inflammatory mediators IL-1β, IL-6, TNFα, iNOS, and COX2 were measured by quantitative real-time RT-PCR (**A–E**). Protein levels of pro-inflammatory mediators were analyzed using ELISA or western blotting (**F–I**). All data are presented as the mean ± SD from three independent experiments. *P < 0.05 for LPS vs. LPS plus NL-1.

Macrophages play critical roles in various inflammatory diseases through release of inflammatory mediators and cytokines such as IL-1β, IL-6, and TNFα. To investigate the role of mitoNEET during inflammatory responses, we analyzed LPS-stimulated expression of cytokines and mediators by RAW264.7 cells in the presence or absence of a mitoNEET inhibitor, mitoNEET Ligand-1 (NL-1), which was derived from glitazones [28] (Fig. 2). We found that NL-1 reduced expression of mRNA encoding pro-inflammatory cytokines IL-1β, IL-6, and TNFα, and of mRNA encoding inflammatory mediators iNOS and COX2, in cells exposed to LPS for 12 h (Fig. 2A–E). This decrease was not seen in control cells (treated with LPS alone). In addition, IL-1β, IL-6, TNFα, iNOS, and COX2 protein levels decreased in the presence of NL-1 (Fig. 2F–I).

**Fig. 3:**
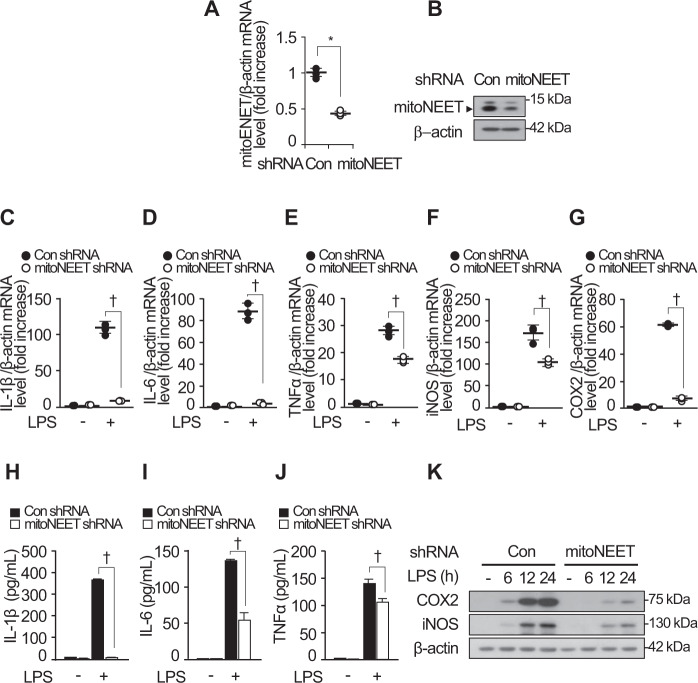
Expression of mitoNEET shRNA decreases the levels of inflammatory mediators produced by RAW264.7 cells. RAW264.7 cells were transfected with control shRNA or mitoNEET shRNA and then subjected to RT-PCR or western blotting to verify downregulation of mitoNEET expression (**A** and **B**). β-actin was used as a loading control. RAW264.7 cells transfected with control shRNA or mitoNEET shRNA were stimulated with LPS (100 ng/mL) or vehicle. Total RNA, cell supernatants, and total protein were harvested 12 h (for mRNA) or 24 h (for protein) later. Expression of mRNA encoding IL-1β, IL-6, TNFα, iNOS, and COX2 was assessed by quantitative real-time RT-PCR (**C–G**), and protein expression was analyzed by ELISA or western blotting (**H–K**). All data are expressed as the mean ± SD from three independent experiments. *P < 0.05 for control shRNA-expressing cells vs. mitoNEET shRNA-expressing cells. ^†^*P* < 0.05 for control shRNA-expressing cells vs. mitoNEET shRNA-expressing cells in the presence of LPS.

To investigate whether LPS-induced expression of mitoNEET alters inflammatory responses, we generated mitoNEET shRNA or control shRNA-expressing cells. Real-time PCR and western blot analyses were performed to assess expression of mitoNEET mRNA and protein, respectively (Fig. 3A, B). Expression of mRNA encoding IL-1β, IL-6, TNFα, iNOS, and COX2 decreased in mitoNEET shRNA-expressing cells compared with control shRNA-expressing cells (Fig. 3C–G). This was also the case for protein expression (Fig. 3H–K). These data indicate that LPS-stimulated expression of mitoNEET is involved in inflammatory responses by macrophages via release of pro-inflammatory cytokines and mediators.

**Fig. 4:**
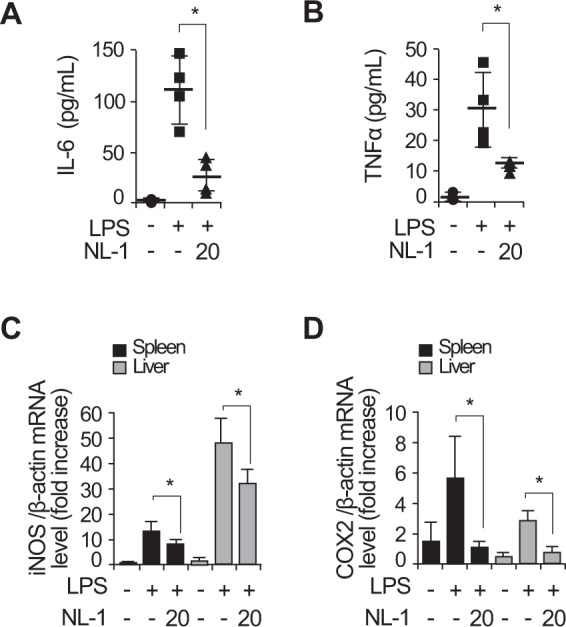
Expression of inflammatory mediators triggered by LPS-induced sepsis was diminished in the presence of a mitoNEET inhibitor. C57BL/6 mice were injected intraperitoneally with vehicle, LPS (20 mg/kg), or LPS plus NL-1 (20 mg/kg). Blood was collected from the right atrium 48 h later, and IL-6 and TNFα levels in serum were measured (**A** and **B**). Total RNA was harvested from the spleen and liver. Expression of mRNA encoding iNOS and COX2 was assessed in the spleen or liver (**C** and **D**) by quantitative real-time RT-PCR. For all real-time PCR analyses, β-actin was used as a control for normalization. All data are expressed as the mean ± SD from three independent experiments. *P < 0.05 for LPS vs. LPS plus NL-1 treatment.

To assess the effect of mitoNEET inhibition during LPS-induced sepsis, we injected NL-1 intraperitoneally into wild-type C57BL/6 mice 12 h prior to injection of LPS. Blood was collected from the right atrium 48 h after LPS injection, and the concentration of IL-6 and TNFα in serum was measured. Control mice received vehicle alone. The levels of IL-6 and TNFα fell markedly in the presence of NL-1 (Fig. 4A, B). Liver- and spleen-mediated immune responses are responsible for clearing bacteria and toxins, but they can also cause inflammation and organ damage [36, 37]. We found that NL-1 reduced expression of mRNA encoding iNOS (Fig. 4C) and COX2 (Fig. 4D) in the spleen and liver of mice with LPS-induced sepsis. Taken together, these data demonstrate that mitoNEET is a key regulator of inflammatory responses during LPS-induced sepsis.

### Inhibition of mitoNEET attenuates LPS-induced oxidative stress and mitochondrial dysfunction

**Fig. 5:**
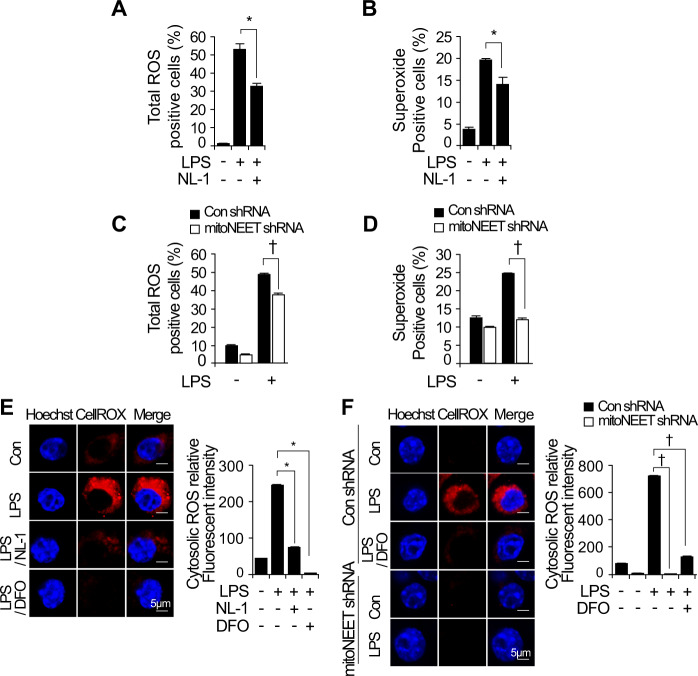
LPS-induced reactive oxygen species and mitochondrial dysfunction are attenuated by inhibition of mitoNEET. RAW264.7 cells expressing control shRNA or mitoNEET shRNA were treated with vehicle, LPS (1 µg/mL), or LPS plus NL-1 (20 µM) for 24 h. Total reactive oxygen species (ROS) and superoxide anions were assayed by flow cytometry using a ROS/Superoxide Detection kit (**A–D**). All data shown are expressed as the mean ± SD from three independent experiments. *P < 0.05 for LPS vs. LPS plus NL-1 treatment; ^†^*P* < 0.05 for control shRNA-expressing cells vs. mitoNEET shRNA-expressing cells in the presence of LPS. *P < 0.05 indicates a significant decrease compared with vehicle; ^†^*P* < 0.05 indicates significant decrease compared with control siRNA. RAW264.7 cells expressing control shRNA or mitoNEET shRNA were treated with vehicle or LPS (1 µg/mL) for 12 h. Cells were then stained with the fluorescent probes Hoechst 33258 (nuclei, blue) and CellROX^®^ Deep Red (total ROS, red). Immunofluorescence images of cells stained with Hoechst 33258 (nuclei, blue) and CellROX^®^ Deep Red (total ROS, red) (**E** and **F**). Scale bar: 5 µm. Fluorescence intensity was measured using image analysis software (ImageJ). All data are expressed as the mean ± SD from three independent experiments. *P < 0.05 for LPS vs. LPS plus NL-1 or DFO. ^†^*P* < 0.05 for control shRNA-expressing cells vs. mitoNEET shRNA-expressing cells in the presence of LPS or vs. LPS plus DFO.

**Fig. 6:**
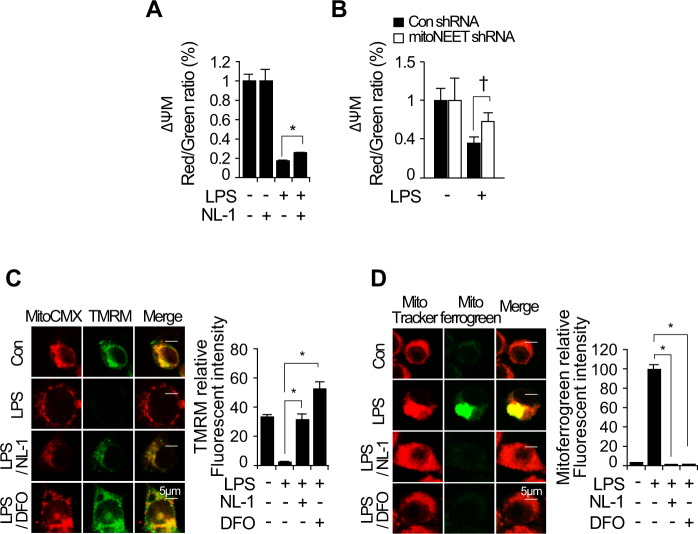
LPS-induced ROS and mitochondrial dysfunction are activated by mitoNEET-mediated iron accumulation. RAW264.7 cells expressing control shRNA or mitoNEET shRNA were treated with vehicle, LPS (1 µg/mL), or LPS plus NL-1 (20 µM) for 24 h. The mitochondrial membrane potential (MMP) was measured by flow cytometry using MitoProbe JC-1 (**A** and **B**). The histogram shows the ratio of JC-1 polymer (red) to JC-1 monomer (green) fluorescence, which is an index of the MMP. A decrease in the red/green ratio indicates depolarization of the mitochondrial membrane. RAW264.7 cells expressing control shRNA or mitoNEET shRNA were treated with vehicle, LPS (1 µg/mL), LPS plus NL-1 (20 µM), or LPS plus DFO (500 µM) for 12 h. Cells were then stained with MitoTracker Red CMXRos, DiOC6(3), (red), and TMRM (tetramethylrhodamine, methyl ester, Perchlorate, green). (**C**). Cells were stained with MitoTracker Deep Red (a mitochondrial marker; red) and Mito-FerroGreen (a marker of mitochondrial Fe2+; green) after treatment (**D**). Immunofluorescence images of MitoTracker Red CMXRos, DiOC6(3), (mitochondria, red), TMRM (green), and MitoTracker Deep Red, Mito-FerroGreen (green) (**C** and **D**). Scale bar: 5 µm. Fluorescence intensity was measured using ImageJ. All data are expressed as the mean ± SD from three independent experiments. *P < 0.05 for LPS vs. LPS plus NL-1 or DFO. ^†^*P* < 0.05 for control shRNA-expressing cells vs. mitoNEET shRNA-expressing cells in the presence of LPS.

Inflammatory processes induce oxidative stress and alter mitochondrial function [12]. When cells are under oxidative stress, mitoNEET acts as a redox-sensitive protein to induce transfer of the [2Fe–2S] cluster in mitochondria and plays a role in production of ROS [25]. Therefore, we analyzed whether inhibiting mitoNEET protects RAW264.7 cells from LPS-induced oxidative stress and mitochondrial dysfunction. Cells were treated with LPS in the presence or absence of NL-1 for 24 h. Next, total ROS and superoxide were assayed by flow cytometry using a ROS/Superoxide Detection kit. LPS-induced total ROS and superoxide fell in the presence of NL-1 (Fig. 5A, B). Furthermore, to investigate whether downregulation of mitoNEET regulates oxidative stress, we stimulated control shRNA- or mitoNEET shRNA-expressing RAW264.7 cells for 12 h with LPS (1 µg/mL), and measured total ROS and superoxide levels by flow cytometry. Total ROS and superoxide levels fell in LPS-treated cells expressing mitoNEET shRNA (Fig. 5C, D). In addition, confocal microscopy clearly demonstrated that NL-1 or mitoNEET shRNA suppressed LPS-induced cytosolic ROS when compared with LPS alone (Fig. 5E, F). Treatment of cells with the iron chelator DFO (deferoxamine) in the presence of LPS showed results comparable to those observed after mitoNEET inhibition by NL-1 or mitoNEET shRNA (Fig. 5E, F). To verify whether inhibition of mitoNEET regulates mitochondrial dysfunction, we examined the mitochondrial membrane potential (MMP), a hallmark of mitochondrial dysfunction. Cells were treated NL-1 in the presence or absence of LPS, and the loss of MMP was measured by flow cytometry using MitoProbe JC-1. Inhibition of mitoNEET rescued LPS-induced depolarization of the mitochondrial membrane (Fig. 6A). LPS-induced loss of MMP was also rescued by mitoNEET shRNA (Fig. 6B). To verify the effects of NL-1 on the MMP, we stained LPS-stimulated RAW264.7 cells with mitochondrial probes MitoTracker Red CMXRos, DiOC6(3), or TMRM (tetramethylrhodamine, methyl ester) in the presence or absence of NL-1. TMRM staining is used widely to monitor MMP. The MMP in LPS-treated cells fell but was rescued by NL-1 (Fig. 6C). DFO showed similar effects. To verify that inhibition of mitoNEET decreases the mitochondrial iron content, we stained mitochondrial iron using the mitochondrial probes Mitochondrial Marker Deep Red and Mito-ferroGreen by, which allow visualization of ferrous ion (Fe^2+^) by confocal microscopy. We found that NL-1 depleted mitochondrial Fe^2+^ in the presence of LPS. Similar results were obtained using DFO (Fig. 6D). Taken together, these data suggest that inhibiting mitoNEET in RAW264.7 cells reduces mitochondrial iron content, thereby preventing oxidative stress and mitochondrial dysfunction during LPS-induced inflammation.

**Fig. 7:**
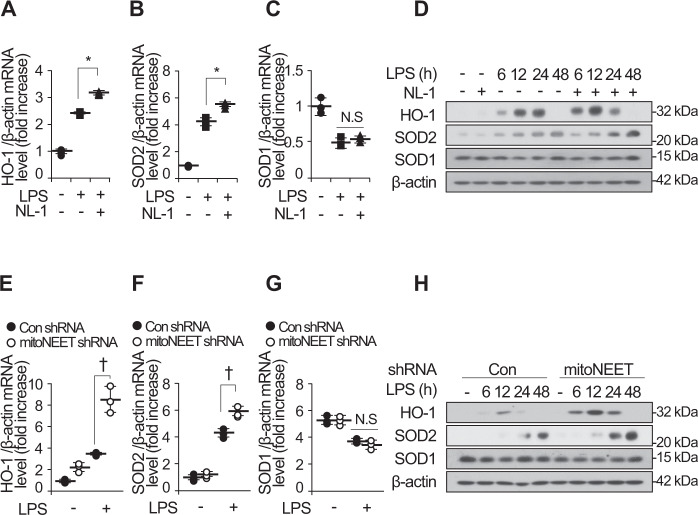
Inhibition of mitoNEET increases expression of antioxidant defense genes. Control shRNA-expressing or mitoNEET-expressing RAW264.7 cells were treated with vehicle, LPS (1 µg/mL), or LPS plus NL-1 (20 µM). Total RNA and protein were harvested 12 h later, followed by measurement of mRNA encoding HO-1, SOD2, or SOD1 by quantitative real-time RT-PCR (**A–C** and **E–G**). Expression of HO-1, SOD2, and SOD1 protein was detected by western blotting (**D** and **H**). All data are expressed as the mean ± SD from three independent experiments. *P < 0.05 for LPS vs. LPS plus NL-1 treatment. ^†^*P* < 0.05 for control shRNA-expressing cells vs. mitoNEET shRNA-expressing cells in the presence of LPS. NS, not significant.

Oxidant-induced injury during inflammatory processes such as sepsis induces organ failure [10]. To confirm the anti-inflammatory effects of NL-1 during LPS-induced oxidative stress, we examined expression of HO-1 (heme oxygenase-1), SOD2 (superoxide dismutase 2), and SOD1 (superoxide dismutase 1) mRNA and protein in LPS-stimulated RAW264.7 cells in the presence or absence of NL-1 (Fig. 7A–D). NL-1 increased expression of HO-1 and SOD2 mRNA and protein, but not that of SOD1 mRNA and protein. Consistent with this, HO-1 and SOD2 mRNA and protein levels in cells expressing mitoNEET shRNA were higher than those in control shRNA-expressing cells (Fig. 7E–H). These results demonstrate that NL-1 or mitoNEET shRNA may attenuate oxidative-induced organ injury during LPS-induced inflammation by upregulating expression of antioxidant-defense genes.

## Discussion

Sepsis is one of the most serious causes of mortality worldwide. There is increasing evidence that oxidative stress plays a major role in organ dysfunction by driving excessive inflammation [13, 38, 39]. Inflammation-induced ROS production promotes dysfunction of mitochondria, a major site of ROS production, thereby activating oxidative stress and generating a self-feeding cycle [11, 40]. Thus, a therapeutic strategy targeting mitochondrial dysfunction has the potential to break this vicious cycle and prevent progression of oxidative stress and sepsis [13]. Recent studies suggest that iron is an essential component of cellular processes such as mitochondrial energy metabolism; however, mitochondrial iron overload is a major cause of mitochondrial damage and ROS [13, 41, 42]. In this study, we demonstrated that inhibiting mitoNEET, a mitochondrial iron regulator, has a protective effect against inflammatory responses during sepsis. MitoNEET, a mitochondrial protein, plays a key role in energy metabolism, iron regulation, and production of ROS by mitochondria [19, 43]. Our data show that inflammatory stimuli, such as LPS, which induce mitochondrial oxidative damage, trigger production of mitoNEET mRNA and protein in various animal models of and in BMDMs (Fig. 1). Furthermore, the LPS-induced NF-kB signaling pathway is involved in induction of mitoNEET expression during inflammatory conditions (Fig. 1H). These data suggest that mitoNEET could play a key role in energy metabolism as well as in inflammation. Interestingly, expression of inflammatory mediators IL-1β, IL-6, TNFα, iNOS, and COX2 decreased in the presence of a mitoNEET inhibitor, NL-1, or upon expression of mitoNEET shRNA, even in the LPS-induced sepsis model (Figs. 2, 3, and 4). MitoNEET was first identified as a redox-sensitive mitochondrial target of the thiazolidinedione (TZD) pioglitazone [28, 29]. Earlier studies observed that overexpression of mitoNEET inhibits mitochondrial iron transport to the matric and reduces ROS-mediated damage [21]. This led to a reduction in the MMP and accumulation of ROS in mouse adipocytes [22]. Conversely, we found that ROS levels, MMP, and iron content were reduced in the presence of NL-1 or mitoNEET shRNA under inflammatory conditions (Figs. 5 and 6). Furthermore, expression of the antioxidant enzyme HO-1 and the mitochondrial MnSOD isoform SOD2 was enhanced in the presence of NL-1 or mitoNEET shRNA after LPS administration; however, expression of SOD1, a major cytoplasmic antioxidant enzyme, was not enhanced. The reason for these different results could be because mitoNEET acts differently according to the redox conditions in a cell. The biophysical properties of mitoNEET involve electron and Fe–S cluster transfer [17, 44, 45]. In a reducing environment, mitoNEET is incapable of [2Fe–2S] cluster transfer; thus accumulation of iron in the mitochondria is abrogated by accelerating loss of the [2Fe–2S] cluster [46]. However, only when cells are under oxidative stress does mitoNEET [2Fe–2S] transfer [2Fe–2S] clusters to apoproteins, and electrons from NADH to oxygen or ubiquinone, in mitochondria [18, 19, 25]. In an oxidizing environment, mitoNEET contributes to oxidative stress and production of superoxide radicals (O_2_^•−^) by transferring iron to the mitochondrial matrix and electrons to oxygen through oxidation of NADH (the electron donor). Recent publications demonstrated that pioglitazone stabilizes the 2Fe–2S cluster and inhibits iron transfer from mitoNEET to mitochondria. Pioglitazone, which shows strong preferential binding to mitoNEET in the oxidized state, may therefore act to alleviate stress caused by Fe overload [25]. These data suggest that iron regulation via targeting of mitoNEET rescues ROS production and mitochondrial dysfunction in the oxidized state [41, 47, 48]. Therefore, our results demonstrate that mitoNEET is a possible therapeutic molecule for mitochondrial dysfunction during inflammatory diseases and sepsis.

## Materials and methods

### Cell culture and reagents

RAW264.7 cells were cultured in Dulbecco Modified Eagle Medium (Life Technologies, Grand Island, NY, USA), 5% fetal bovine serum, 100 units/mL penicillin, and 100 mg/mL streptomycin under an atmosphere of 95% air and 5% CO_2_ at 37 °C. Bone marrow-derived macrophages (BMDMs) from C57BL/6 mice were isolated and differentiated as described previously [49]. Briefly, bone marrow cells (3 × 10^7^ cells) were cultured in macrophage-differentiation medium with GM-CSF at 37 °C for 7 days. The adherent macrophages were detached from culture dishes by treatment with 5% EDTA in PBS, followed by scraping with a sterile cell scraper. The resuspended cells were then directly seeded on cell culture plates for other experiments.

mitoNEET inhibitor, NL-1, was purchased from (Merck Millipore, Billerica, MA, USA, 475825). Deferoxamine mesylate salt (DFO) was purchased from (Sigma-Aldrich, St Louis, MO, D9533). Lipopolysaccharides from Escherichia coli O26:B6, *γ*-irradiated, BioXtra, suitable for cell culture (Sigma-Aldrich, St Louis, MO, L2654).

### Animal experiments

Animal care and use for all experiments were approved from the animal facilities at University of Ulsan (SWC-14-012). C57BL/6 mice were purchased from ORIENT BIO Inc (Busan, Korea). CLP-induced polymicrobial sepsis and fibrin clot experiment were performed as previously described [50]. Using sterile conditions, the fibrin clot containing *E. coli* or *S. aureus* (1 × 10^8^ CFU) was placed within the peritoneal cavity of C57BL/6 mice. Lipopolysaccharides from *E. coli* 0127:B8—purified by phenol extraction were purchased from (Sigma-Aldrich, St Louis, MO, L3129) for LPS-induced sepsis. mitoNEET inhibitor, NL-1 (20 mg/kg), was administrated to C57BL/6 mice 12 h before LPS (20 mg/kg) injection (pre-administration).

### Western immunoblotting and enzyme-linked immunosorbent assay

Total protein isolation and Western blotting were performed as described previously [ref]. The blots were incubated with antibody an anti-CISD1 (1:2000) (Protein Tech, 16006-1-AP), an anti-β-actin (1:5000) (Sigma-Aldrich, St Louis, MO, A5441), an anti-phospho-IkBα (1:1000) (Santa Cruz Biotechnology, sc-8404), an anti-IkBα (1:1000) (Santa Cruz Biotechnology, sc-847) and an anti-COX2 (1:1000) (Cayman chemical, 160106), an anti-iNOS (1:1000) (Santa Cruz Biotechnology, sc-650) and an anti-HO-1 (1:3000) (Enzo Life Sciences, ADI-SPA-896), an anti-SOD2 (1:3000) (Santa Cruz Biotechnology, sc-30080), an anti-SOD1 (1:1000) (Santa Cruz Biotechnology, sc-11407) and anti-NLRP3/NALP3 (1:1000) (Adipogen, AG-20B-0014-C100), an-anti-P2X7R (1:3000) (Alomone Labs, APR-004) in TBST overnight at room temperature. The blots were incubated with an anti-secondary antibody (1:5000) in TBST, immunoblots were detected by SuperSignal^®^ West Pico Chemiluminescent Substrate (Pierce) and visualized after exposure to X-ray film.

Mouse IL-1β (R&D systems, Minneapolis, MN, USA, DY401), IL-6 (R&D systems, Minneapolis, MN, USA, DY406), and TNFα (R&D systems, Minneapolis, MN, USA, DY410) were measured from cell culture supernatant of BMDMs and RAW264.7 cells using enzyme-linked immunosorbent assay (ELISA).

### Quantitative real-time reverse transcription–polymerase chain reaction (qRT-PCR)

Total RNA was isolated TRIzol reagent (Invitrogen, Life technologies, Carlsbad, CA), Reverse transcription was performed using SuperScript™ III First-Strand Synthesis System (Invitrogen, Carlsbad, CA). Real-time quantitative PCR was conducted using iQ SYBR Green Supermix (Bio-Rad, Hercules, CA). Triplicate samples per condition were analyzed on an Applied Biosystems StepOnePlusTM Real-Time PCR System using absolute quantification settings. The primers sequences were as follows: mouse mitoNEET (forward: 5′-CAA GGC TAT GGT GAA TCT TCA G-3′ and reverse: 5′-GTG CCA TTC TAC GTA AAT CAG-3′), mouse β-actin (forward: 5′-GAT CTG GCA CCA CAC CTT CT-3′ and reverse: 5′-GGG GTG TTG AAG GTC TCA AA-3′). Mouse IL-1β (forward: 5′-TTG ACG GAC CCC AAA AGA TG-3′ and reverse: 5′-AGA AGG TGC TCA TGT CCT CA-3′), mouse IL-6 (forward: 5′-GAG GAT ACC ACT CCC AAC AGA CC-3′ and reverse: 5′-AAG TGC ATC ATC GTT GTT CAT ACA-3′), mouse TNFα (forward: 5′- GCC TCT TCT CAT TCC TGC TTG-3′ and reverse: 5′-CTG ATG AGA GGG AGG CCA TT-3′), mouse IL-6 (forward: 5′-GAG GAT ACC ACT CCC AAC AGA CC-3′ and reverse: 5′- AAG TGC ATC ATC GTT GTT CAT ACA-3′), and mouse COX2 (forward: 5′-CAA GGG AGT CTG GAA CAT TG-3′ and reverse: 5′-ACC CAG GTC CTC GCT TAT GA-3′), mouse iNOS (forward: 5′-AAC GGA GAA CGT TGG ATT TG-3′ and reverse: 5′-CAG CAC AAG GGG TTT TCT TC-3′), mouse HO-1 (forward: 5′-CGC CTT CCT GCT CAA CAT T-3′ and reverse: 5′-TGT GTT CCT CTG TCA GCA TCA C-3′), mouse SOD2 (forward: 5′- ATG GTG GGG GAC ATA TT-3′ and reverse: 5′-GAA CCT TGG ACT CCC ACA GA -3′). Amplification of cDNA started with 10 min at 95 °C, followed by 40 cycles of 15 s at 95 °C and 1 min at 60 °C.

### Construction of mitoNEET shRNA-expressing cells

The mitoNEET shRNA and the nonspecific control shRNA (Sigma-Aldrich, St Louis, MO) were transfected into RAW264.7 cells using transfection reagents (Promega, Madison, WI, USA) according to the manufacturer’s protocol. The sequences of mouse mitoNEET shRNA were as follows: 5′-CCG GCG TAG GAC CTC TGA TCA TCA ACT CGA GTT GAT GAT CAG AGG TCC TAC GTT TTT TG-3′. The expression of mitoNEET and β-actin in stable cells was measured.

### Flow cytometry for total reactive oxygen species (ROS) and superoxide measurement

Cells were treated with vehicle, LPS, LPS plus NL-1, or LPS plus DFO (desferoxamine). Total ROS and superoxide were detected using ROS-ID^®^ total ROS/Superoxide Detection kit (Enzo Life Sciences, Farmingdale, NY, USA, ENZ-51010) according to the protocol of the manufacturer. Measurements were performed on a FACS Calibur (Becton Dickinson, San Jose, CA, USA) flow cytometer.

### Flow cytometry for mitochondrial membrane potential measurements

To measure the mitochondrial membrane potential, cells were stained using the MitoProbe JC-1 staining kits (MitoProbe™ JC-1 Assay Kit for Flow Cytometry, Thermo Fisher Scientific, Carlsbad, CA, USA, M34152) according to the protocol of the manufacturer. Measurements were performed on a FACSCalibur (Becton Dickinson, San Jose, CA, USA) flow cytometer (Ex = 488 nm and Em = 590 nm for JC-1 aggregates; Ex = 488 nm and Em = 529 nm for JC-1 monomers). Histogram of the percent of JC-1 red/green ratio calculated the relative ratio of red (JC-1 polymer) against green (JC-1 monomer) fluorescence.

### Confocal microscopy

RAW264.7 cells were seeded at 1 × 10^5^ cells per well on coverslips in 24-well plates and treated with reagents. After reagent treatment, media was removed by washing with PBS and cells were incubated with serum free media containing 5 µM Cell ROX red (CellROX^®^ Deep Red Reagent for oxidative stress detection, Invitrogen, Life Technologies, Carlsbad, CA, C10422) or 200 nM Mitotracker red CMXRos (Invitrogen, Life Technologies, Carlsbad, CA, M7512) and 500 nM TMRM (tetramethylrhodamine, methyl ester, Perchlorate, Invitrogen, Life Technologies, Carlsbad, CA, T668) or 100 nM Mitotracker red (Mitochondrial marker deep red, Invitrogen, Life Technologies, Carlsbad, CA, M22426) and 5 µM Mito-FerroGreen (Dojindo Laboratories, Kumamoto, Japan, M489) for 30 min at 37 °C in the dark. Then, cells fixed for 20 min in 4% formaldehyde, rinsed 3 times in PBS. A nuclear counterstaining was made with a solution of 1 µg/mL Hoechst 33258 stain for 5 min and mounting on a slide Fluorescence Mounting Medium (DAKO North America Inc, Carpinteria, CA, United States, S3023). Olympus FV1000 MPE microscope was used to acquire images.

### Statistical analysis

All results were confirmed in at least three independent experiments; data from one representative experiment are shown. Quantitative data are shown as means ± standard deviation and significance of statistical analysis was determined with two-tailed, unpaired Student’s *t*-test. *P*-values <0.05 were considered significant.

## Supplementary information

Reproducibility Checklist

## Data Availability

All data needed to evaluate the conclusions in the paper are present in the paper. Additional data related to this paper may be requested from the corresponding author.
